# The Behaviour of Sequestra

**Published:** 1909-09-25

**Authors:** 


### Surgery.

### THE BEHAVIOUR OF SEQUESTRA.

The life history of a sequestrum from the time of its formation to that of its ultimate absorption or its removal by the surgeon varies according to a number of factors. The chief of these are the nature of the infective process originally responsible for the disease and the locality which is affected. A sequestrum is essentially a portion of dead bone remaining in the body.

Large sequestra are usually the result of acute inflammation in a long bone, in which, as in osteomyelitis, a greater or lesser portion of the shaft undergoes necrosis. The bone dies in cases of osteomyelitis, because it is deprived of its blood supply by a double action; the virulence of the infection causes thrombosis of the branches of its nutrient artery and the superficial blood supply is interfered with by the stripping-off of its periosteum. The whole shaft may form one solid sequestrum, or if the infection is very acute it may be broken up into small pieces by pathological processes and discharged through an external wound if there be one. The details of the following case, which is an example of this, were supplied to the writer by the medical officer of a big-game shooting expedition. The patient, one of the baggage-porters, was an African negro, aged 38. He was stabbed during a fight in the greater tuberosity of the right humerus with a septic instrument. The wound suppurated and discharged pus without intermission. On the tenth day small fragments of dead bone were seen in the discharge. After the first few days, except for occasional pyrexia, his general health did not appear to be affected. The small sequestra continued to come away, and it was not until several weeks after the date of the infliction of the injury that the expedition returned to its base. There was a small *x*-ray apparatus in the town, and a skiagram was taken. It was then seen that the upper three-quarters of the shaft of the humerus were gone, and the periosteum was much thickened; but that it did not yet form a definite bony involucrum was shown by the fact that the upper arm was not rigid. It was obvious that the shaft had been gradually extruded in the form of minute sequestra. The after-history was interesting. In two months the discharge ceased and a complete new shaft was gradually formed from the periosteum, giving the patient a useful arm with no obvious deformity, except that on careful palpation the surface of the bone could be felt to be slightly irregular in outline. Such a good result is rarely obtained in this country.

Acute inflammation in bone may also produce a small sequestrum, if it is confined to the superficial aspect as in a localised suppurative periostitis. The death of the bone is due in this case to the stripping-up of the periosteum only, since the superficial parts of a long bone depend for their nutrition almost entirely on the periosteal blood supply.

The first effect of the presence of a sequestrum is to produce an osteo-blastic reaction in the overlying periosteum; and thus a protecting layer of new bone is formed, circular in the case of a central sequestrum, flat in that of a laminar. As soon as the involucrum is formed, the sequestrum acts as an irritant and a perforation appears in the involucrum, which sooner or later makes its way to the surface. Occasionally a very small sequestrum is absorbed completely without the formation of a cloaca, but this is the exception. The cloaca is Nature's method of getting rid of the dead bone.

Much may be done by surgical intervention. But it is highly important to remember that no good can be done by operating before the sequestrum is separated. In deciding this a skiagram affords the greatest possible help by displaying the outlines of the sequestrum. Occasionally, however, one is misled even by a good skiagram. This happened to the writer recently. The patient, a boy aged 13, had suffered from osteomyelitis of the left femur. He had, when seen, an involucrum extending from the condyles to the lesser trochanter and a small cloaca on the outer side of the leg at the junction of the lower and middle thirds. A skiagram was taken and a large sequestrum was seen, which appeared to be quite loose. A long incision was made on the outer side of the limb and a large groove chiselled in the involucrum. An attempt was made to remove the sequestrum, but this was found to be impossible, because it was still fixed at its upper end. The wound was accordingly closed. Six weeks later another skiagram was taken, and, on the evidence given by this, a second operation was performed. The sequestrum was then successfully removed, without fracturing the involucrum, which was firm and bony. In removing the sequestrum from the humerus or femur, it is well to wait until the involucrum is firm; otherwise the limb may telescope from want of support. Many plans have been devised to overcome this—*e.g.* the introduction of animals' bones or celluloid or ivory rods, but the simplest and most effective is to apply an extension to the limb until such time as the new bone is strong enough to resist the contraction of the muscles. This difficulty does not arise in the leg or forearm, where the second bone acts as a most efficient splint.

Tuberculous disease of bone also causes the formation of sequestra. But the infection being subacute and the process one of caries, in which the bone is gradually replaced by granulation tissue, rather than necrosis, in which it undergoes sudden death owing to lack of nutrition, the sequestra produced are usually small and broken up *ab initio*. Moreover, they tend to make their way to the surface and discharge themselves without the formation of an involucrum. Occasionally, when a central focus of tuberculosis occurs in a long bone, some compensatory osteosclerosis may take place at the periphery; but this is not in the true sense of the word an involucrum. Syphilitic disease of the skull causes sequestrum formation of a characteristic type, the sequestra being circular or irregular in outline and tabular in form.

